# The GEM-handle as convenient labeling strategy for bimodal single-domain antibody-based tracers carrying ^99m^Tc and a near-infrared fluorescent dye for intra-operative decision-making

**DOI:** 10.3389/fimmu.2023.1285923

**Published:** 2023-11-15

**Authors:** Noemi B. Declerck, Celine Huygen, Lukasz Mateusiak, Marcus C. M. Stroet, Sophie Hernot

**Affiliations:** Molecular Imaging and Therapy Laboratory (MITH), Vrije Universiteit Brussel (VUB), Brussels, Belgium

**Keywords:** nanobody, bimodal tracer, hybrid tracer, fluorescence imaging, gamma-probing, intraoperative imaging, cancer surgery

## Abstract

Intra-operative fluorescence imaging has demonstrated its ability to improve tumor lesion identification. However, the limited tissue penetration of the fluorescent signals hinders the detection of deep-lying or occult lesions. Integrating fluorescence imaging with SPECT and/or intra-operative gamma-probing synergistically combines the deep tissue penetration of gamma rays for tumor localization with the precision of fluorescence imaging for precise tumor resection. In this study, we detail the use of a genetically encoded multifunctional handle, henceforth referred to as a GEM-handle, for the development of fluorescent/radioactive bimodal single-domain antibody (sdAb)-based tracers. A sdAb that targets the urokinase plasminogen activator receptor (uPAR) was engineered to carry a GEM-handle containing a carboxy-terminal hexahistidine-tag and cysteine-tag. A two-step labeling strategy was optimized and applied to site-specifically label IRDye800CW and ^99m^Tc to the sdAb. Bimodal labeling of the sdAbs proved straightforward and successful. ^99m^Tc activity was however restricted to 18.5 MBq per nmol fluorescently-labeled sdAb to prevent radiobleaching of IRDye800CW without impeding SPECT/CT imaging. Subsequently, the *in vivo* biodistribution and tumor-targeting capacity of the bimodal tracer were evaluated in uPAR-positive tumor-bearing mice using SPECT/CT and fluorescence imaging. The bimodal sdAb showed expected renal background signals due to tracer clearance, along with slightly elevated non-specific liver signals. Four hours post-injection, both SPECT/CT and fluorescent images achieved satisfactory tumor uptake and contrast, with significantly higher values observed for the anti-uPAR bimodal sdAb compared to a control non-targeting sdAb. In conclusion, the GEM-handle is a convenient method for designing and producing bimodal sdAb-based tracers with adequate *in vivo* characteristics.

## 1 Introduction

Cancer surgery remains central in the curative treatment of solid tumors. Yet, specific and accurate intra-operative tumor lesion identification and margin delineation remain challenging. Unnoticed lesions and incorrect delineations can lead to tumor recurrence or iatrogenic tissue damage. Positive resection margins, occurring in 5-35% of operated patients, significantly increase the risk of local cancer relapse, impact the aggressiveness of post-surgical chemotherapy, and decrease overall and disease-specific survival rates (1). Over the last decade, fluorescence-guided imaging has emerged as the front-runner in intra-operative tumor imaging. It has demonstrated its efficacy to provide real-time visualization of tumor lesions without disrupting the surgical field or workflow (2–6). However, the limited tissue penetration of even near-infrared fluorescent signals remains a constraint for identifying occult tumor lesions beyond a few millimeters of depth. This can be overcome by combining fluorescence detection with pre-operative nuclear imaging and/or intra-operative gamma-probing. This combination offers an optimal complementary relationship between the high sensitivity of gamma-ray detection for the localization of hidden tumor lesions and the visual cues provided by fluorescent signals for precise tumor resection (7, 8). In recent years, non-targeted ICG-[^99m^Tc]Tc-nanocolloids have successfully improved sentinel lymph node mapping through their bimodal nature compared to the separate modalities; thus advocating for a combined use of both modalities (9–12).

To further implement bimodal cancer imaging in clinical practice, bimodal tracers based on tumor marker-directed targeting moieties are essential to adequately identify and resect lesions. For their design, both labels are preferentially attached onto the same targeting moiety to ensure consistent pharmacokinetic behavior. The simplest approach involves random conjugation of both labels separately onto the targeting moiety (13–16). However, this results in heterogeneous end products and may negatively impact the functionality or pharmacokinetic behavior of small targeting moieties (17, 18). A more controlled approach employs trivalent platforms that combine a fluorophore, a radiolabeling site and an attachment site on a single scaffold (19–28). This approach ensures precise and consistent spatial positioning of the labels relative to each other. The trivalent platforms can then be attached to the targeting moiety either randomly or in a site-specific manner. However, the production of trivalent platforms is labor-intensive as it involves a multi-step synthesis. More recently, platforms have been explored that utilize the fluorophore itself as the linking scaffold between the targeting moiety and the chelator, reducing the overall size of the construct (28–30).

**Figure 1:**
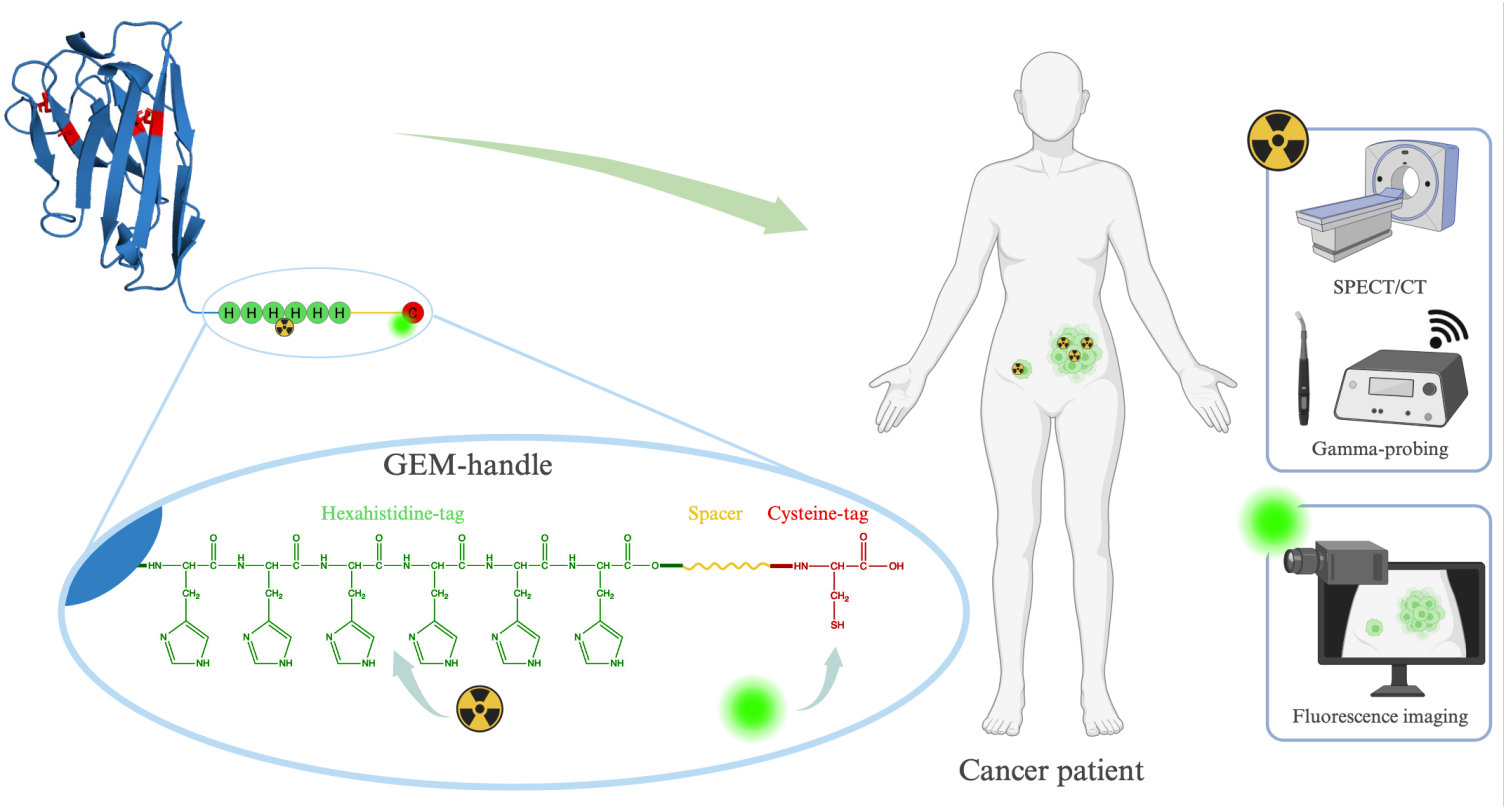
Schematic representation of our GEM-handle engineered into a sdAb and its potential applications.

In this study, we introduce an alternative and more convenient approach for designing bimodal tracers, namely the Genetically Encoded Multifunctional-handle (GEM-handle). The amino acid sequence of peptide- or protein-based targeting moieties can be readily engineered to encode additional amino acid motives, which enable labeling through various site-directed chemistries. Since these sites are inherent to the overall structure of the molecule, they ensure a consistent positioning of the labels on the targeting moiety. Our GEM-handle comprises two labeling sites, a hexahistidine-tag and a cysteine-tag, separated by a 14-amino acid spacer (**Figure 1**). The cysteine-tag allows for labeling with any maleimide-functionalized near-infrared fluorophore of interest, while the hexahistidine-tag exclusively permits labeling with technetium-99m (^99m^Tc) (31). Notably, ^99m^Tc is a low-energy gamma-ray emitter routinely employed in clinical practice for both pre-operative SPECT/CT imaging and intra-operative gamma-probing. This makes this isotope well-suited for the intended purpose of bimodal tracers.

Our GEM-handle leverages the carboxy-terminal tags we routinely incorporate for site-specific labeling on single-domain antibodies (sdAbs). sdAbs are ideal small antibody fragments derived from *camelid* heavy-chain antibodies for the development of targeted bimodal tracers due to their distinct advantages: remarkable target specificity, compact size and ease of engineering. Unlike antibodies, which remain in circulation for days and tend to accumulate non-specifically, sdAbs enable high-contrast imaging within hours after injection and with minimal background because of their rapid renal clearance. Moreover, in comparison with peptides, sdAbs serve as a platform technology that allows straightforward generation of sdAbs targeting almost any biomarker of interest, along with predictable pharmacokinetics. Previous preclinical and clinical investigations involving sdAb-based tracers for fluorescent, nuclear or bimodal imaging have shown the potential of these targeting moieties (3, 27, 32–39). In this paper, we report on our GEM-handle labeling approach for achieving bimodal labeling of sdAb-based tracers, focusing on a sdAb previously designed to target the urokinase plasminogen activator receptor (uPAR) (40). Furthermore, we evaluate its *in vivo* biodistribution and effectiveness in targeting tumors in a subcutaneous mouse model.

## 2 Materials and methods

### 2.1 GEM-handle modified sdAb production

The anti-human/canine uPAR sdAb uPAR15 was previously generated and validated preclinically by Mateusiak et al. (40). uPAR15 and the control sdAb R3b23 were cloned into a pHEN25 plasmid coding for a carboxy-terminal tail encoding a hexahistidine-tag and a cysteine-tag separated by a rigid 14 amino acid linker based on the hinge region of human IgA1 (**Figure 1**) (39, 41). The sdAb-GEM constructs were produced and purified according to a previously described method (39, 41). Briefly, sdAb-GEM constructs were expressed in *E. coli* and purified from periplasmic extracts using immobilized metal affinity chromatography followed by size exclusion chromatography (SEC). After purification, uPAR15-GEM and R3b23-GEM presented in monomeric form (with the cysteine-tag linked to glutathione) and dimeric form (two sdAbs linked by their cysteine-tags). Both forms were collected as separate fractions.

### 2.2 Two-step bimodal labeling of the sdAb-GEM

#### 2.2.1 Reduction of the cysteine-tag and fluorescence labeling

As a first step in the bimodal labeling procedure, maleimide-cysteine chemistry was employed to label uPAR15-GEM and R3b23-GEM site-specifically with IRDye800CW (Li-COR Biosciences; Nebraska, USA). SdAb-GEM (3 mg) was incubated with a 90- or 180-fold molar excess of 2-mercaptoethylamine (Thermo Fisher Scientific; Massachusetts, USA) for dimer or monomer fractions, respectively, and EDTA (5 mM, Sigma-Aldrich; Missouri, USA) in PBS (2.5 mL, pH 7.4; Thermo Fisher Scientific) at 37°C for 90 min. A PD-10 desalting column (Cytiva; Hoegaarden, Belgium) was equilibrated with ammonium acetate buffer (25 mL, 0.2 M, pH 6.0; Sigma-Aldrich) and subsequently, a buffer exchange was performed by applying the reduced sdAb-GEM and eluting with ammonium acetate (3.5 mL 0.2 M, pH 6.0). The collected sdAb-GEM was transferred to a Vivaspin column (Vivaspin 25,000 MWCO HY; Sartorius; Göttingen, Germany) and concentrated to a volume of 0.5 mL. Then, the reduced sdAb-GEM was incubated with a 5-fold molar excess of IRDye800CW-maleimide (20 mg/mL in DMSO) and EDTA (5 mM, 1 mL in 0.2 M ammonium acetate, pH 6.0) in a light-protected vial at 37°C for 120 min. Purification of the sdAb-GEM-IRDye800CW from the excess IRDye800CW was performed via SEC on a Superdex Increase 75 10/300 GL (Cytiva) with PBS (pH 7.4) as a running buffer (0.8 mL/min). The sdAb-GEM and dye concentration of the IRDye800CW-labeled compounds was calculated through absorption measurement at 280 nm for the sdAb and at 774 nm for IRDye800CW using spectrophotometry (Nanodrop 2000; Thermo Fisher Scientific). To attribute for the absorption of IRDye800CW at 280 nm, the value measured at 280 nm was corrected by 3% of the value at 774 nm before calculation. The degree of labeling of the constructs was determined as the concentration of IRDye800CW to sdAb-GEM (42).

Quality control (QC) of each sdAb-GEM-IRDye800CW was performed via SEC on a Superdex Increase 75 10/300 GL (Cytiva) with PBS (pH 7.4) as a running buffer (0.8 mL/min). Detection was performed through absorption at 280 nm and 774 nm. The purity of the sdAb-GEM-IRDye800CW was considered adequate at 95% or higher, determined as the percentual area-under-the-curve (%AUC) of the sdAb-GEM-IRDye800CW peak on the QC SEC profile.

#### 2.2.2 Radiolabeling with ^99m^Tc and quality control

As a second step, uPAR15-GEM-IRDye800CW and R3b23-GEM-IRDye800CW were labeled with [[^99m^Tc]Tc(H_2_O)_3_(CO)_3_]^+^ through histidine-tricarbonyl-chemistry (31, 43). [^99m^Tc]TcCO_4_^-^ (1 mL, from a ^99^Mo/^99m^Tc generator, Drytec, GE Healthcare; Illinois, USA) was added to a lyophilized kit (IsoLink™, Covidien; St Louis, USA) and the sealed vial was heated to 100˚C in a water bath for 30 min to enable the conversion to [[^99m^Tc]Tc(H_2_O)_3_(CO)_3_]^+^. After conversion, the pH of the kit was adjusted to 6.5 – 6.8 with 1 M HCl. Subsequently, sdAb-GEM-IRDye800CW (2 nmol or 12 nmol of protein for *in vitro* and *in vivo* experiments, respectively) were incubated with [[^99m^Tc]Tc(H_2_O)_3_(CO)_3_]^+^ (activities ranging between 0 and 185 MBq/nmol) in PBS (250-500 µl, pH 7.4) at 50°C for 90 min. At 0, 1, 3 and 6 h post-radiolabeling, absorption and fluorescent signal of the sdAb-GEM-[^99m^Tc]Tc(CO)_3_-IRDye800CW compounds were assessed using SEC and fluorescence scanning respectively. For SEC, Tween 80 (25 µL, 0.1% m/v in PBS, Sigma-Aldrich) was added to the sdAb-GEM-[^99m^Tc]Tc(CO)_3_-IRDye800CW samples and 250 µL was injected on a Superdex 75 10/300 GL column (Cytiva) with PBS (pH 7.4) as running buffer (0.5 mL/min). Detection was performed through absorption measurements at 280 nm and 774 nm, as well as through gamma-counting (WIZARD^2^ 2480 Gamma Counter; PerkinElmer; Massachusetts, USA). Absorption of IRDye800CW in sdAb-GEM-[^99m^Tc]Tc(CO)_3_-IRDye800CW samples was compared to absorption of the non-radioactively labeled sdAb-GEM-IRDye800CW to assess the fluorophore stability. For fluorescence scanning, a 1:50 dilution of the sdAb-GEM-[^99m^Tc]Tc(CO)_3_-IRDye800CW samples was applied in triplicate to a 96-well plate along with unlabeled sdAb-GEM-IRDye800CW as positive control. The post-radiolabeling fluorescent signal was compared to the signal of the positive control using the Odyssey scanner 9120 (Li-COR). Additionally, to ensure the temperature and kit buffer did not negatively affect the fluorescent signal of the compounds, radiolabeling was performed at 37 and 21°C compared to 50°C, and the sdAb-GEM-IRDye800CW constructs were incubated in a decayed kit under the above-described conditions.

For further *in vivo* use, sdAb-GEM-[^99m^Tc]Tc(CO)_3_-IRDye800CW were purified via NAP 5 columns (Cytiva) and eluted with Tween-PBS (1 mL, 0.01%, pH 7.4) to remove any free [[^99m^Tc]Tc(H_2_O)_3_(CO)_3_]^+^ and were then filtered (Merck Millipore 0.22 µm syringe filter; Merck & Co.; New Jersey, USA) to eliminate possible aggregates. Before and after purification, the radiochemical purity of the sdAb-GEM-[^99m^Tc]Tc(CO)_3_-IRDye800CW was assessed via instant thin-layer chromatography (iTLC) using acetone as running buffer. sdAb-GEM-[^99m^Tc]Tc(CO)_3_-IRDye800CW remained at the baseline and ^99m^Tc eluted to the top of the iTLC paper. sdAb-GEM-[^99m^Tc]Tc(CO)_3_-IRDye800CW with a radiochemical purity above 95% after purification was adequate for *in vivo* experiments.

### 2.3 Hydrophobic interaction chromatography

uPAR15-GEM, uPAR15-GEM-IRDye800CW, uPAR15-GEM-[^99m^Tc]Tc(CO)_3_, and uPAR15-GEM-[^99m^Tc]Tc(CO)_3_-IRDye800CW (70 µg) were prepared in ammonium sulfate (1 mL, 0.5 M, Sigma-Aldrich). HIC was performed on a HiTrap Butyl HP 1 mL column (Cytiva) using 0.5 M ammonium sulfate and MilliQ H_2_O as running buffers (1 mL/min). The starting buffer was 100% ammonium sulfate (0.5 M). After 8 min, gradient elution started for 12 min until the running buffer was 100% MilliQ water which then ran for an additional 10 min. Detection was performed through absorption measurements at 280 nm and 774 nm, as well as gamma-counting.

### 2.4 *In vivo* biodistribution and tumor targeting of bimodal sdAb

All animal studies were performed according to the European Directive 2010/63/EU and received approval from the Ethical Commission for Animal Experimentation of the Vrije Universiteit Brussel (project nr. 21-272-13). Female Crl : NU-Foxn1nu mice were purchased from Charles River at 6 weeks old (18 - 25 g). The mice were group housed in individually ventilated cages at 19 to 24°C and 40 to 60% humidity with 4 mice per cage. A light/dark cycle of 14/10 h was implemented. Low-fluorescence pellet food (Teklad 2016, Basis Global Technologies; Illinois, USA) and water were provided *ad libitum*. After tumor inoculation and growth to 200 – 500 mm^3^, the mice were randomly allocated to the experimental and control groups (4 mice per group) by a blinded laboratory technician. Upon inclusion in the experiment, starting from the tumor inoculation, all mice were inspected daily by assessment of behavior, appearance, and tumor growth. Tumors with a size above 100 mm^3^ were measured every two to three days. The humane endpoints applied in this study were 1) a tumor size above 1500 mm^3^ or a tumor ulceration above 10% of the tumor volume, 2) a body condition score of 1, and 3) a physical appearance or abnormal behavior indicative of pain or sickness. All mice in the study were killed through cervical dislocation. Injections, imaging, and killing of the mice were carried out under isoflurane anesthesia (5% induction, 2% maintenance, 1.0 L/min O_2_).

#### 2.4.1 Longitudinal assessment of the *in vivo* biodistribution and tumor targeting capacity of anti-uPAR bimodal sdAb

HT-29 cells (ATCC; Virginia, USA) were cultured in McCoy’s medium supplemented with fetal bovine serum, glutamine, and penicillin/streptavidin at 37°C and 5% CO_2_. Female Crl : NU-Foxn1nu mice (n = 4, N = 16) were subcutaneously inoculated with 2 x 10^6^ uPAR-positive HT-29 cells in the right flank and allowed to grow till 200 – 500 mm^3^. 2 nmol uPAR15-GEM-[^99m^Tc]Tc(CO)_3_-IRDye800CW or R3b23-GEM-[^99m^Tc]Tc(CO)_3_-IRDye800CW (23 ± 2 MBq) was intravenously injected via the tail vein. SPECT/CT imaging was performed 1, 4, and 8 h post-injection (p.i.) and fluorescence imaging was performed 1, 4, 8, and 24 h p.i. For each bimodal tracer, a group of mice was killed at 1 and 24 h p.i. and relevant organs and tissues were collected for *ex vivo* analysis through immediate fluorescence imaging and ^99m^Tc gamma-counting post-dissection. *Ex vivo* radioactive uptake values for the tumor and relevant organs were decay-corrected and are described as %ID/g.

#### 2.4.2 Imaging protocol and analysis

SPECT/CT imaging was performed for 25 min using the Vector^+^ microSPECT/CT system from MILabs (Houten, Netherlands). The system was fitted with a rat/mouse 75 pinhole collimator (1.5 mm). Spiral mode SPECT scans were performed over 6 bed positions (200 s/position). CT scans were performed immediately after the SPECT scans (60 kV, pixel size = 80 µm). SPECT scans were reconstructed with the SPECT-Rec software (MILabs) after acquisition (subsets = 2, iterations = 4, voxel size = 0.4 mm) and paired with the corresponding CT scans. Further image analysis was handled in the Amide and OsiriX software. 3D regions-of-interest (ROIs) were allocated on the SPECT images for the tumor and the %ID/cc in the ROIs was determined.

*In vivo* and *ex vivo* fluorescence imaging was performed using the FluoBeam800 (Fluoptics; Grenoble, France) in a dark room. White light images were obtained under normal room light. Acquisition was performed on the raw data setting with exposure times of 25 – 300 ms. Fluorescent images were analyzed in ImageJ (Fiji). 2D ROIs were drawn onto the white light images around the tumor, the muscle of the opposing hind leg or the relevant organs. The ROIs were transferred onto the fluorescent images for mean fluorescent intensity (MFI) determination. *In vivo* tumor-to-background ratios (TBRs) were calculated as the ratio of the tumor MFI to the contralateral muscle MFI.

### 2.5 Statistical analysis

*In vivo* fluorescence TBRs of the experimental and control groups were compared using an unpaired student t-test per imaging modality. *In vivo* and *ex vivo* overall uptake values and absolute MFI values were compared using an unpaired student T-test. All statistical analyses were performed using GraphPad Prism (version 8.4.3., GraphPad Software; California, USA) with p values: *p<0.05, **p<0.01, ***p<0.001, and ****p<0.0001. All data is presented as mean ± SD and the graphical representations of the data were made with GraphPad Prism 8.4.3.

## 3 Results

### 3.1 Fluorescence labeling of sdAb-GEM

**Figure 2:**
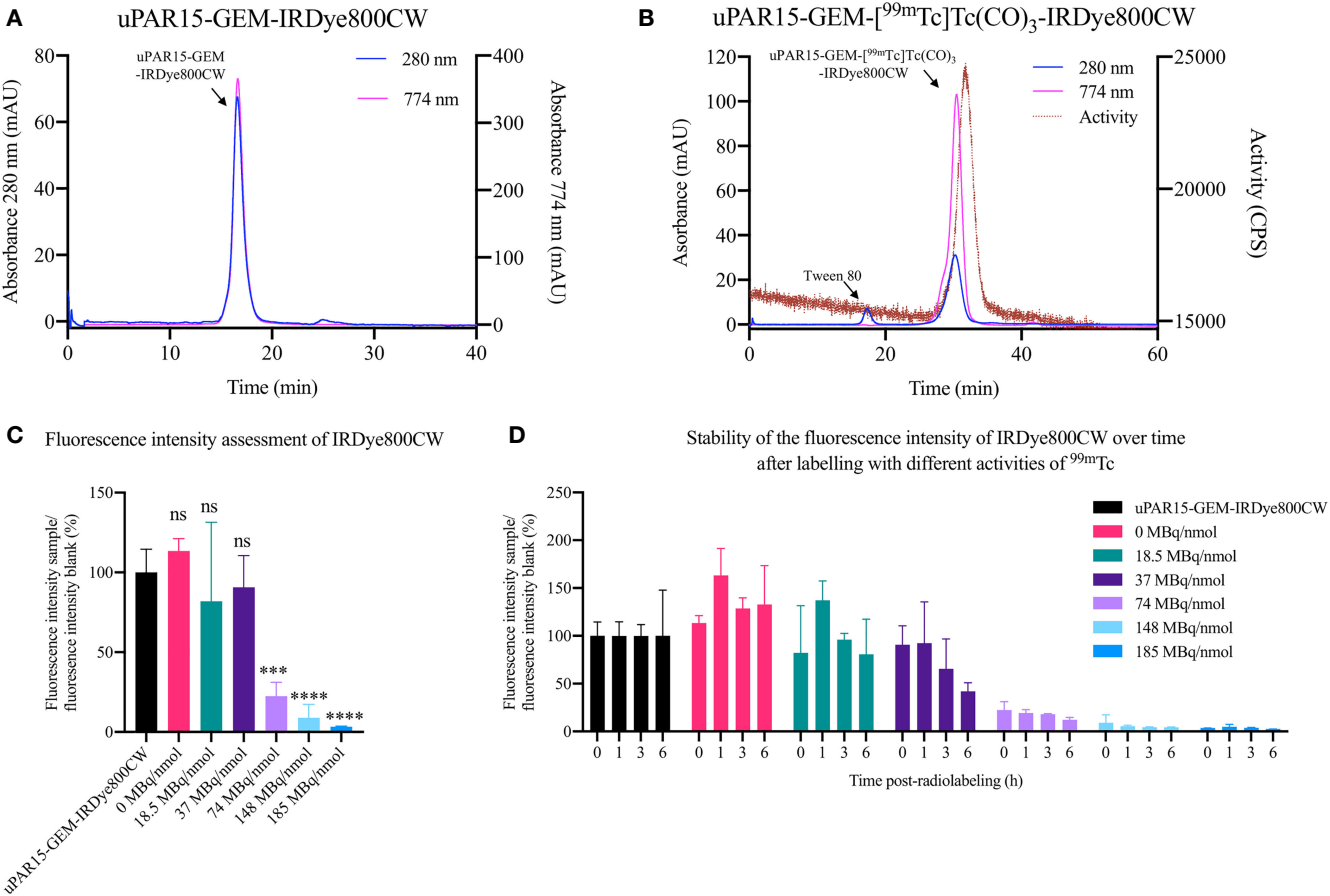
QC via SEC for uPAR15-GEM-IRDye800CW after fluorescence labeling **(A)** and for uPAR15-GEM-[^99m^Tc]Tc(CO)_3_-IRDye800CW after radiolabeling **(B)**. Fluorescence intensity assessment of uPAR15-GEM-IRDye800CW 0 h post-radiolabeling **(C)** and 0, 1, 3, and 6 h post-radiolabeling **(D)** with increasing amounts of [[^99m^Tc]Tc(H_2_O)_3_(CO)_3_]^+^ from 0 to 185 MBq/nmol. All results are presented as mean ± SD of the relative percentual MFI of the radio-labeled samples to the blank, uPAR15-GEM-IRDye800CW. (n = 4; ns ≥ 0.05, p*** < 0.001, p**** < 0.0001).

For this study, the sdAb-GEM were labeled with IRDye800CW and [[^99m^Tc]Tc(H_2_O)_3_(CO)_3_]^+^ via a two-step labeling strategy utilizing respectively the cysteine-tag and hexahistidine-tag embedded in the GEM-handle. Firstly, uPAR15-GEM and R3b23-GEM were successfully labeled with IRDye800CW as indicated by the two coinciding peaks at 280 nm (sdAb-GEM) and 774 nm (IRDye800CW) on the SEC QC profiles (**Figure 2A**; **Supplementary Figure 1A**). Based on the %AUC, the purity of uPAR15-GEM-IRDye800CW and R3b23-GEM-IRDye800CW was respectively 97.8% and >99.9%. The degree of labeling of the respective sdAb-GEM-IRDye800CW constructs was 0.9 and 0.8. A minor fraction of unconjugated sdAb-GEM remained in the mixture, which could not be further purified through SEC due to the minor molecular weight difference.

### 3.2 Radiolabeling optimization of sdAb-GEM-IRDye800CW

In the second step, the sdAb-GEM-IRDye800CW were labeled with ^99m^Tc via their His-tag, achieving a radiochemical purity of > 87.0% as determined by iTLC analysis. For subsequent *in vivo* applications, the compounds were further purified to achieve a radiochemical purity exceeding 99% (**Figure 2B**; **Supplementary Figure 1B**). Nevertheless, it was noted that the fluorescent signal of the sdAb-GEM-IRDye800CW was influenced by the ^99m^Tc-labeling, with the extent of the impact being dependent on the added amount of radioactivity (priorly, it had been confirmed that the incubation temperature and the composition of the buffer in the lyophilized Isolink kit for ^99m^Tc labeling did not impact the fluorescence of sdAb-GEM-IRDye800CW (data not shown)). The fluorescent signal of 2 nmol of uPAR15-GEM-[^99m^Tc]Tc(CO)_3_-IRDye800CW remained similar to the reference uPAR15-GEM-IRDye800CW after radiolabeling with 18.5 MBq/nmol in 250 µL and remained stable for at least 6h after incubation at room temperature. At 37 MBq/nmol the fluorescent signal was preserved until 1h post-radiolabeling; however, a gradual decrease in signal was observed at later time points (**Figures 2C, D**). For conditions above 37 MBq/nmol, an almost complete loss of fluorescent signal was observed immediately after radiolabeling (**Figures 2C, D**). This corroborates with the SEC profiles of the uPAR15-GEM-[^99m^Tc]Tc(CO)_3_-IRDye800CW samples showing a decline in absorption at 774 nm similar to the decline in fluorescent signal after radiolabeling (**Supplementary Figure 2**). Interestingly, the radiolabeling could be upscaled by increasing the mass of sdAb-GEM-IRDye800CW and the reaction volume. As such, incubating 12 nmol of sdAb-GEM-IRDye800CW with 222 MBq (18.5 MBq/nmol) in 500 µL proved possible with preservation of the fluorescent signal of the bimodal tracer (**Figure 2B**; **Supplementary Figure 1B**). This is necessary for *in vivo* studies.

**Figure 3:**
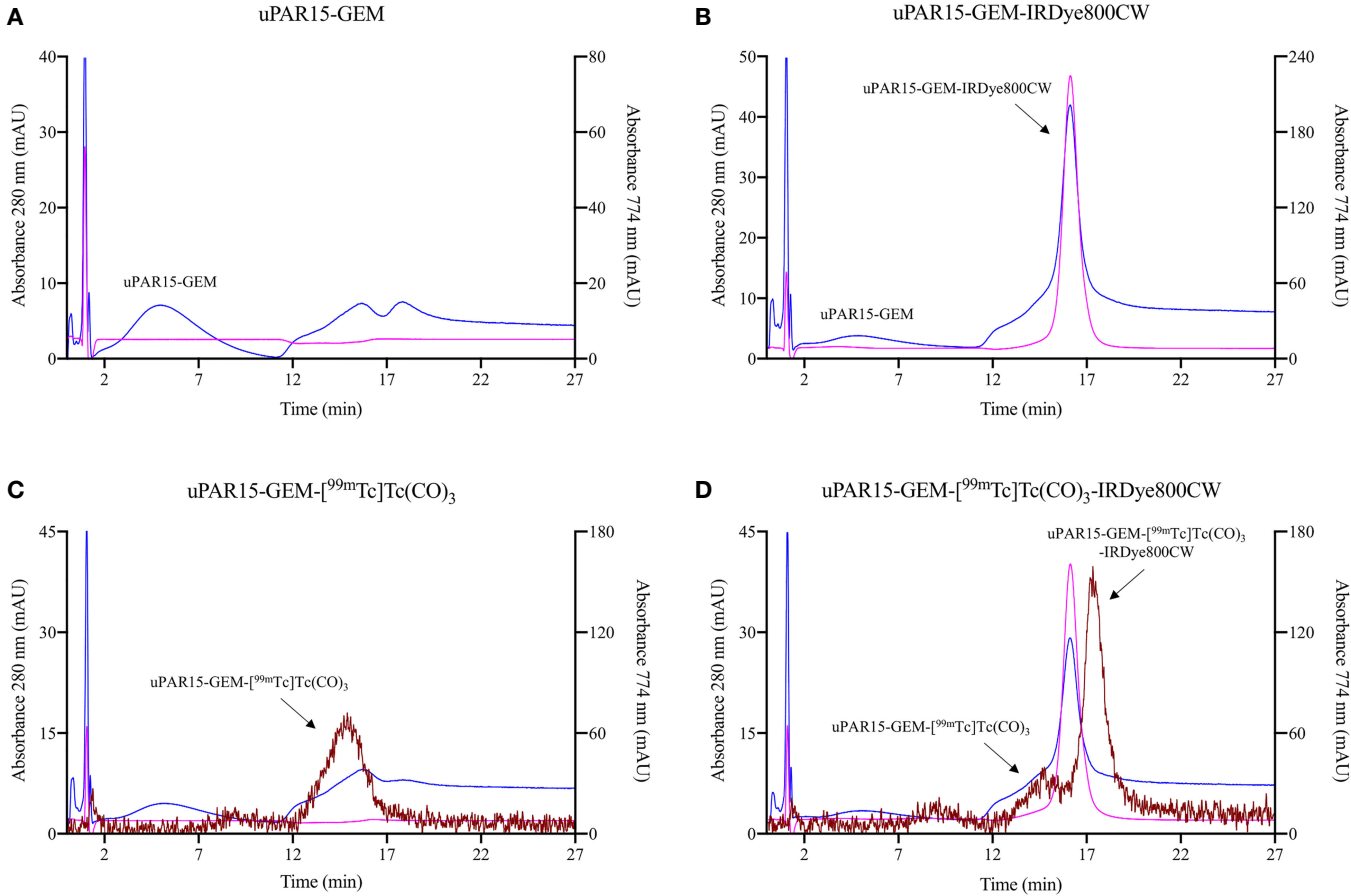
HIC profiles for uPAR15-GEM **(A)**, uPAR15-GEM-IRDye800CW **(B)**, uPAR15-GEM-[^99m^Tc]Tc(CO)_3_ **(C)** and uPAR15-GEM-[^99m^Tc]Tc(CO)_3_-IRDye800CW **(D)**. Detection via absorption of sdAb at 280 nm (blue), of IRDye800CW at 774 nm (pink), and gamma detection for ^99m^Tc (brown).

Next, HIC was used to verify whether [[^99m^Tc]Tc(H_2_O)_3_(CO)_3_]^+^ exhibited any preferential labeling towards the minor fraction of unconjugated sdAb-GEM that remained after fluorescent labeling. On HIC profiles, distinct retention times were observed for uPAR15-GEM, uPAR-GEM-IRDye800CW and uPAR15-GEM-[^99m^Tc]Tc(CO)_3_ (**Figures 3A–C**). Evaluation of the radioactive HIC profile for the uPAR15-GEM-[^99m^Tc]Tc(CO)_3_-IRDye800CW sample revealed two peaks, corresponding respectively with ^99m^Tc-labeled uPAR15-GEM and uPAR15-GEM-IRDye800CW (**Figure 3D**). As the ratio of these peaks corresponds to the degree of labeling (0.8-0.9), it indicates no structural bias in labeling either uPAR15-GEM or uPAR15-GEM-IRDye800CW.

### 3.3 Longitudinal *in vivo* biodistribution and tumor targeting capacity of anti-uPAR bimodal sdAbs

**Figure 4:**
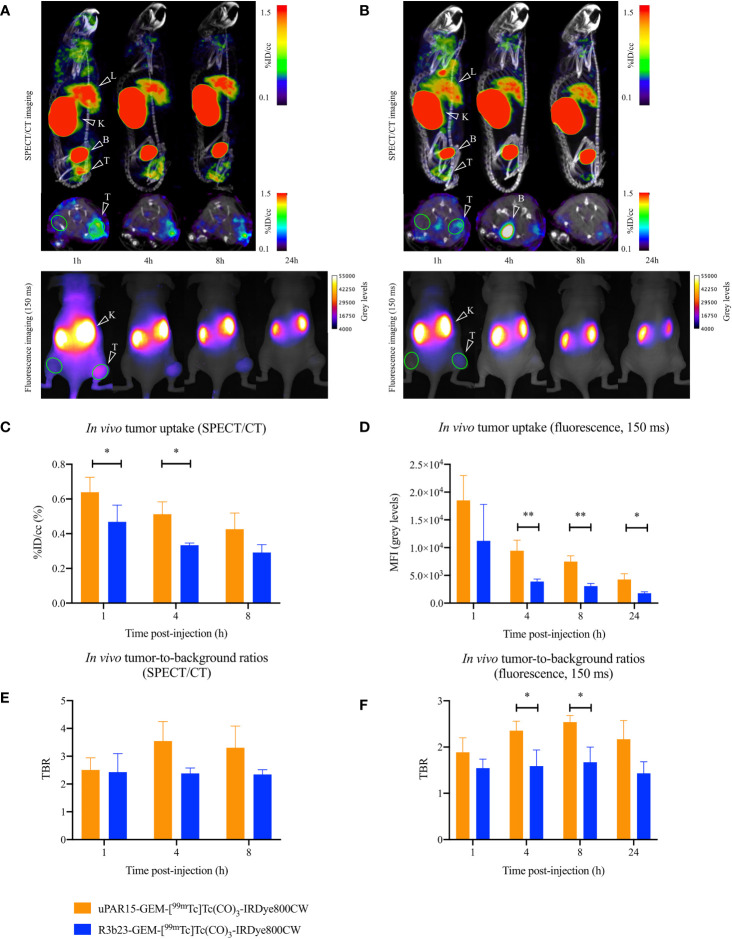
Sagittal and transversal SPECT/CT (top), and dorsal 2D fluorescent (bottom) images of the same mouse carrying a subcutaneous uPAR-positive tumor in the right flank 1, 4, 8 (SPECT/CT and fluorescence imaging) and 24 h (fluorescence imaging) p.i. of uPAR15-GEM-[^99m^Tc]Tc(CO)_3_-IRDye800CW **(A)** or R3b23-GEM-[^99m^Tc]Tc(CO)_3_-IRDye800CW **(B)**. Tumor (T), liver (L), kidneys (K) and bladder (B) are indicated on the images. ROI used to quantitatively delineate the tumor and contralateral muscle are indicated in green. *In vivo* SPECT/CT tumor uptake **(C)**, *in vivo* SPECT/CT TBR-values **(E)**, *in vivo* tumor MFI-values **(D)**, and *in vivo* fluorescent TBR-values **(F)** 1, 4, 8 (SPECT/CT and fluorescence imaging) and 24 h (fluorescence imaging) p.i. Results presented as mean ± SD (n = 4; p* < 0.05, p** < 0.01).

**Figure 5:**
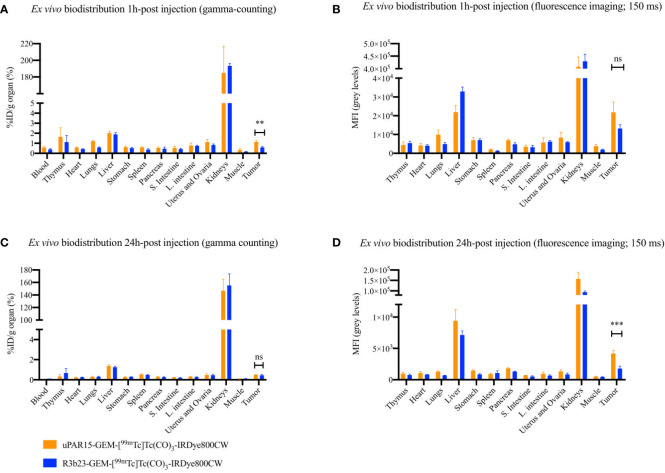
*Ex vivo* uptake-values for uPAR15-GEM-[^99m^Tc]Tc(CO)_3_-IRDye800CW and R3b23-GEM-[^99m^Tc]Tc(CO)_3_-IRDye800CW at 1 h **(A)** and 24 h **(C)** p.i. *Ex vivo* MFI-values for uPAR15-GEM-[^99m^Tc]Tc(CO)_3_-IRDye800CW and R3b23-GEM-[^99m^Tc]Tc(CO)_3_-IRDye800CW at 1 h **(B)** and 24 h **(D)** p.i. Results are presented as mean ± SD (n = 4; ns ≥ 0.05, p** < 0.01, p*** < 0.001).

Finally, the biodistribution and tumor-targeting capacity of uPAR15-GEM-[^99m^Tc]Tc(CO)_3_-IRDye800CW were evaluated *in vivo* in HT-29 tumor-bearing mice and compared to the non-targeting R3b23-based compound. SPECT/CT and fluorescent images indicate that both uPAR15- and R3b23-GEM-[^99m^Tc]Tc(CO)_3_-IRDye800CW were rapidly eliminated from the circulation via the kidneys (188 ± 24%ID/g at 1h p.i.) and, to a much lesser extent, the liver (1.9 ± 0.2%ID/g at 1h p.i.). The compounds showed significant excretion through urine, yet a substantial portion remained in the kidneys for at least 24 h p.i. (150 ± 18%ID/g) (**Figures 4A, B**; **Supplementary Figure 3**). Minimal uptake was seen in other organs. These observations were confirmed by *ex vivo* analysis at 1 h and 24 h p.i. (**Figure 5**; **Supplementary Figure 4**).

At the level of the tumor, uptake of uPAR15-GEM-[^99m^Tc]Tc(CO)_3_-IRDye800CW was visible on SPECT/CT and fluorescence images as soon as 1 h p.i. and up to 8 h p.i. Although the %ID/cc and MFI of uPAR15-GEM-[^99m^Tc]Tc(CO)_3_-IRDye800CW in the tumor was lower than expected, its uptake was still higher than for R3b23-GEM-[^99m^Tc]Tc(CO)_3_-IRDye800CW. At 1 h p.i., some non-specific tumor accumulation of R3b23-GEM-[^99m^Tc]Tc(CO)_3_-IRDye800CW was still present, but the difference between the bimodal labeled uPAR and R3b23 sdAb became more evident at 4 h p.i. with tumor values of respectively 0.51 ± 0.07%ID/cc and 0.33 ± 0.01%ID/cc (p*<0.05), based on quantitative analysis of SPECT/CT images (**Figures 4C, E**). Also on the fluorescent images, tumor MFI (9458 ± 1883 a.u. vs 3895 ± 439 a.u.; p**<0.01) and TBR-values (2.4 ± 0.2 vs 1.6 ± 0.3; p*<0.05)) were statistically higher at that time point for uPAR15-GEM-[^99m^Tc]Tc(CO)_3_-IRDye800CW (**Figures 4D, F**).

## 4 Discussion

In this study, we explored the GEM-handle as a convenient method for designing combined nuclear and fluorescent tracers and evaluated its potential for the development of sdAb-based bimodal tracers. Due to the genetic encoding of the GEM-handle into the sequence of the targeting moiety, it is directly linked to the structure of the protein at a specific position and requires no separate synthesis. Additional advantages of the GEM-handle are the combination with simple site-specific labeling chemistry guaranteeing consistent label positioning and the interchangeability of the fluorophore, which stands in contrast to trivalent platforms that necessitate comprehensive redesign and synthesis for fluorophore substitution.

The labeling of sdAbs, which carried the GEM-handle, with IRDye800CW and subsequently ^99m^Tc, proved straightforward and successful. However, the amount of ^99m^Tc activity that could be used for radiolabeling had to be restricted to preserve the tracer’s fluorescent signal. Hernandez et al. previously demonstrated that cyanine-based dyes are susceptible to radiobleaching and that this effect depends on the type of radiation and activity dosage (44). Although we observed an activity-dependent effect of ^99m^Tc on IRDye800CW, the extent of the effect was unexpected given the lower radiation energy of ^99m^Tc compared to isotopes such as ^111^In, ^68^Ga, or ^121^Bi. To the best of our knowledge, this problem has not been described previously by other research groups combining cyanine-based fluorophores with ^99m^Tc, warranting further investigation (9–11, 29). Hernandez et al. proposed the addition of scavengers, such as ascorbic acid, to prevent radiobleaching. However, these interfere with the redox reaction of the [^99m^Tc]Tc-tricarbonyl chemistry and adding scavengers post-radiolabeling proved futile as fluorescence was already compromised at that moment. We nevertheless demonstrated a linear up-scalability of the radiolabeling without effect on the fluorescence signal by increasing the tracer’s mass and the reaction volume. A molar activity of 18.5 MBq/nmol enables *in vivo* studies in mice with sufficient tracer mass and radioactivity for fluorescent and radioactive tumor detection (12 nmol labeled with 222 MBq, for 6 mice). This molar activity is also clinically relevant as full-body SPECT/CT imaging and sentinel lymph node gamma-probing require respectively 370 – 1110 MBq and 9.25 – 18.5 MBq per patient (45–48), while 1 -10 mg (65-650 nmol) of fluorescent tracer is likely to be needed for intraoperative fluorescence detection.

The typical *in vivo* biodistribution of sdAb-based tracers is characterized by rapid renal clearance, leading to background signals mainly concentrated in the kidneys and bladder. This efficient clearance coupled with fast target recognition facilitates high-contrast imaging within 1 h p.i (27, 34, 36, 49–52). The bimodal sdAb-GEM tracers presented in this study exhibit a comparable biodistribution profile except for a slightly elevated liver accumulation (approximately 2%ID/g). Furthermore, the targeted bimodal tracer only achieved sufficient tumor contrast 4 h p.i. instead of 1 h p.i. The hydrophobic nature of IRDye800CW, known for its binding to serum proteins and necrotic tissues, is thought to contribute to the non-specific liver accumulation (17, 53–55). In comparison, the [^111^In]In‐MSAP.2Rs15d sdAb- compound previously tested by Debie et al. did not show non-specific liver accumulation, most likely due to the use of a more hydrophilic Cy5-based fluorophore (27). Towards the future, exploration of alternative near-infrared fluorescent dyes possessing improved *in vivo* pharmacokinetic behavior, enhanced radiostability, and a structure that does not interfere with the tricarbonyl chemistry could further enhance the potential of GEM-based bimodal tracers (52–54).

A limitation of this study was the relatively low tumor uptake values seen compared to the uptake values observed in the study conducted by Mateusiak et al., describing the generation and validation of the uPAR15 sdAb (40). This could possibly be explained by the use of a different tumor cell line (human colorectal HT-29 tumor cells versus human glioma U-87 cells). Consequently, we obtained smaller effect sizes, contributing to increased uncertainty in the statistical analysis. A direct comparison between uPAR15-GEM-IRDye800CW, uPAR15-GEM-[^99m^Tc]Tc(CO)_3_, and uPAR15-GEM-[^99m^Tc]Tc(CO)_3_-IRDye800CW in the same tumor model would be required to further assess the impact of the GEM-handle on the *in vivo* targeting of sdAb-based tracers. It remains however important to note that since this uPAR15 sdAb only recognizes the human homolog of uPAR, the murine uPAR expression on tumor-associated stromal cells in the tumor microenvironment does not contribute to the tracer’s uptake, hereby underestimating the total tumor accumulation potential in a human situation (40, 56).

The GEM-handle employed in this study consists of a hexahistidine-tag and a cysteine-tag separated by an amino acid spacer. The inclusion of a hexahistidine-tag in sdAbs (and other recombinant proteins) initially serves purification purposes (57, 58), however, it also offers the advantage of easy radiolabeling with ^99m^Tc through tricarbonyl chemistry (31). ^99m^Tc proves to be an ideal radioisotope for the design of bimodal sdAb tracers given its wide availability via ^99^Mo/^99m^Tc-generators, its half-life aligning well with the blood half-life of sdAbs (49), its relatively low radiation energy profile enhancing the safety for both patients and personnel, and its routine use for gamma-probing in the operating theatre, meaning all hardware, protocols and experience is available. Most often chelators, e.g. HYNIC and MAG3, are employed in clinic to prepare ^99m^Tc-labeled radiopharmaceuticals (59–63). Nevertheless, several compounds in clinical studies (64–66), including sdAbs (NCT 04483167, NCT 04040686) (67–69), make use of ^99m^Tc-tricarbonyl chemistry, showing the potential of this strategy for clinical translation. It is a relatively fast labeling procedure, a lyophilized kit is available, and it can be used in combination with any temperature-stable compound.

The inclusion of a cysteine-tag within the GEM-handle provides the ability for site-specific labeling using any maleimide-functionalized fluorophore of interest. The widespread use of this chemical method ensures ready availability of such fluorophores. A drawback of the cysteine-tag is that it leads to a reduction in sdAb production yield and necessitates a reduction before fluorescence labeling (41). Enhancements to the described GEM-handle approach could involve the integration of alternative site-specific labeling motives, such as those based on enzymes or unnatural amino acids (70, 71).

In conclusion, the GEM-handle is a convenient and fast method for designing and producing bimodal sdAb-based tracers, as well as any other tracer generated through fermentation or synthetic production. Further improvement of the GEM-design and conjugated fluorescent labels will increase its potential towards radiostability, good *in vivo* biodistribution and high contrast tumor imaging.

## Data availability statement

The raw data supporting the conclusions of this article will be made available by the authors, without undue reservation.

## Ethics statement

The animal study was approved by Ethical Commission for Animal Experimentation of the Vrije Universiteit Brussel. The study was conducted in accordance with the local legislation and institutional requirements.

## Author contributions

NBD: Conceptualization, Formal Analysis, Funding acquisition, Investigation, Methodology, Writing – original draft, Writing – review & editing. CH: Investigation, Writing – review & editing. LM: Investigation, Writing – review & editing. MCMS: Conceptualization, Investigation, Writing – review & editing. SH: Conceptualization, Funding acquisition, Methodology, Resources, Supervision, Writing – review & editing.

## Supplementary material

Supplementary Figure 1 Quality control of R3b23-GEM-[^99m^Tc]Tc(CO)_3-_IRDye800CW.

Supplementary Figure 2 SEC profiles of uPAR15-GEM-[^99m^Tc]Tc(CO)_3-_IRDye800CW radiolabeled with 0 - 185 MBq/nmol.

Supplementary Figure 3 2D ventral fluorescent images of tumor-bearing mice 1, 4, 8 and 24 h p.i.

Supplementary Figure 4 Ex vivo fluorescent images of relevant organs and tissues and TOR analysis.
