# Supplementary material for: The GEM-handle as convenient labeling strategy for bimodal single-domain antibody-based tracers carrying 99mTc and a near-infrared fluorescent dye for intra-operative decision-making
Source: Front Immunol. 2023 Nov 15;14:1285923. doi: 10.3389/fimmu.2023.1285923 (PMC10684908; doi:10.3389/fimmu.2023.1285923)
Supplement: Supplementary Figure 1 — Quality control of R3b23-GEM-[99mTc]Tc(CO)3-IRDye800CW. [file DataSheet_1.pdf]

Supplementary Material

The GEM-handle as convenient labeling strategy for bimodal single-domain antibody-based tracers carrying ^{99m}Tc and a near-infrared fluorescent dye for intra-operative decision-making

Noemi B. Declerck[1], Celine Huygen[1], Lukasz Mateusiak[1], Marcus C.M. Stroet[1], Sophie Hernot[1*]

[1]Molecular Imaging and Therapy laboratory (MITH), Vrije Universiteit Brussel (VUB), Brussels, Belgium

*** Correspondence:**
Prof. dr. ir. Sophie Hernot
Sophie.Hernot@vub.be

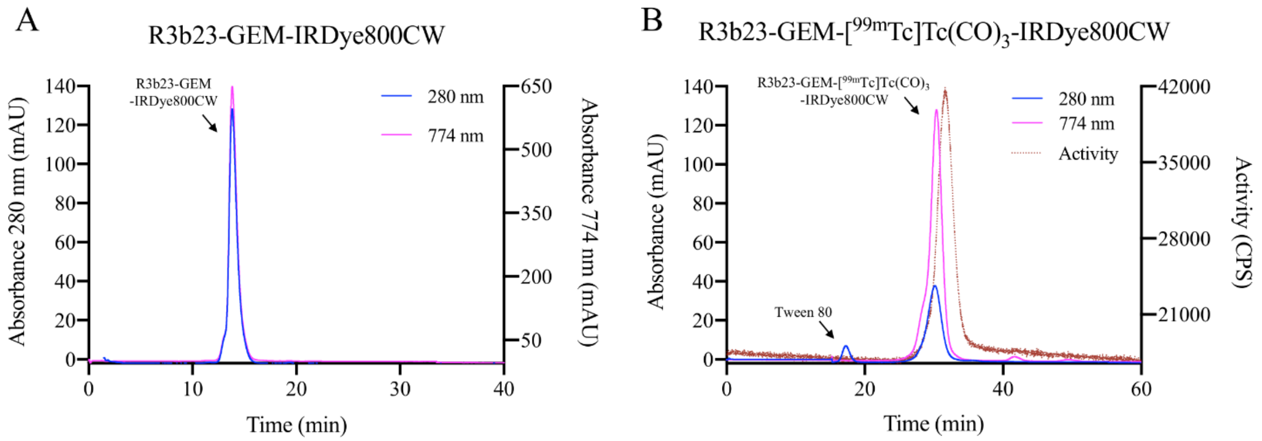

Supplementary Figure 1: QC via SEC for R3b23-GEM-IRDye800CW after fluorescence labelling (**A**) and R3b23-GEM-[^{99m}Tc]Tc$(CO)_3$-IRDye800CW after radiolabeling (**B**).

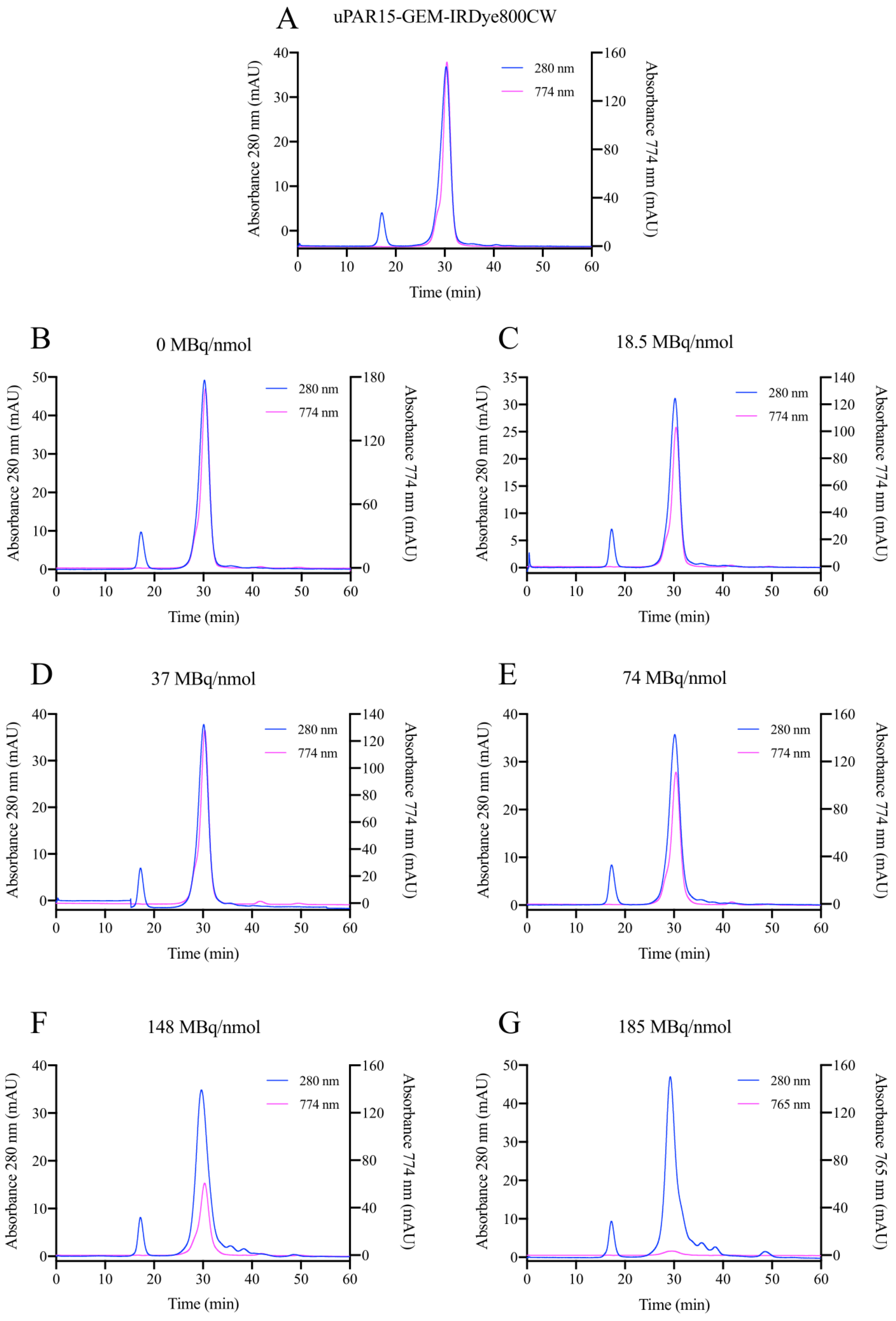

Supplementary Figure 2: SEC profiles for uPAR15-GEM-IRDye800CW (**A**) and uPAR15-GEM-[^{99m}Tc]Tc$(CO)_3$-IRDye800CW radiolabeled with 0 – 185 MBq/nmol activity (**B-G**).

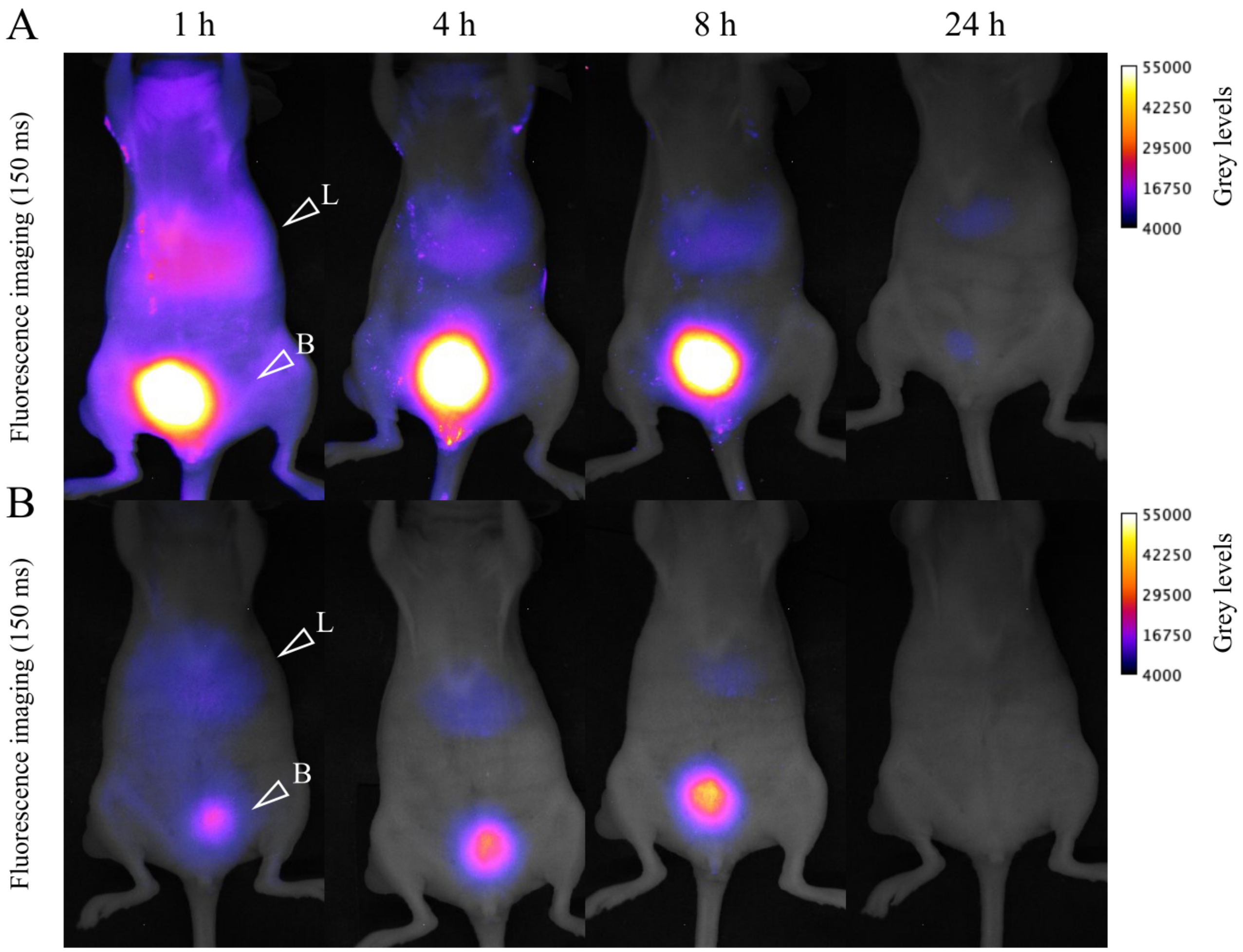

Supplementary Figure 3: Ventral 2D fluorescent images of a mouse carrying a subcutaneous uPAR-positive tumour in the right flank 1, 4, 8 and 24 h post-injection with uPAR15-GEM-[^{99m}Tc]Tc(CO)$_3$-IRDye800CW (**A**) and non-targeted R3b23-GEM-[^{99m}Tc]Tc(CO)$_3$-IRDye800CW (**B**). Liver (L) and bladder (B) are indicated on the first fluorescent images.

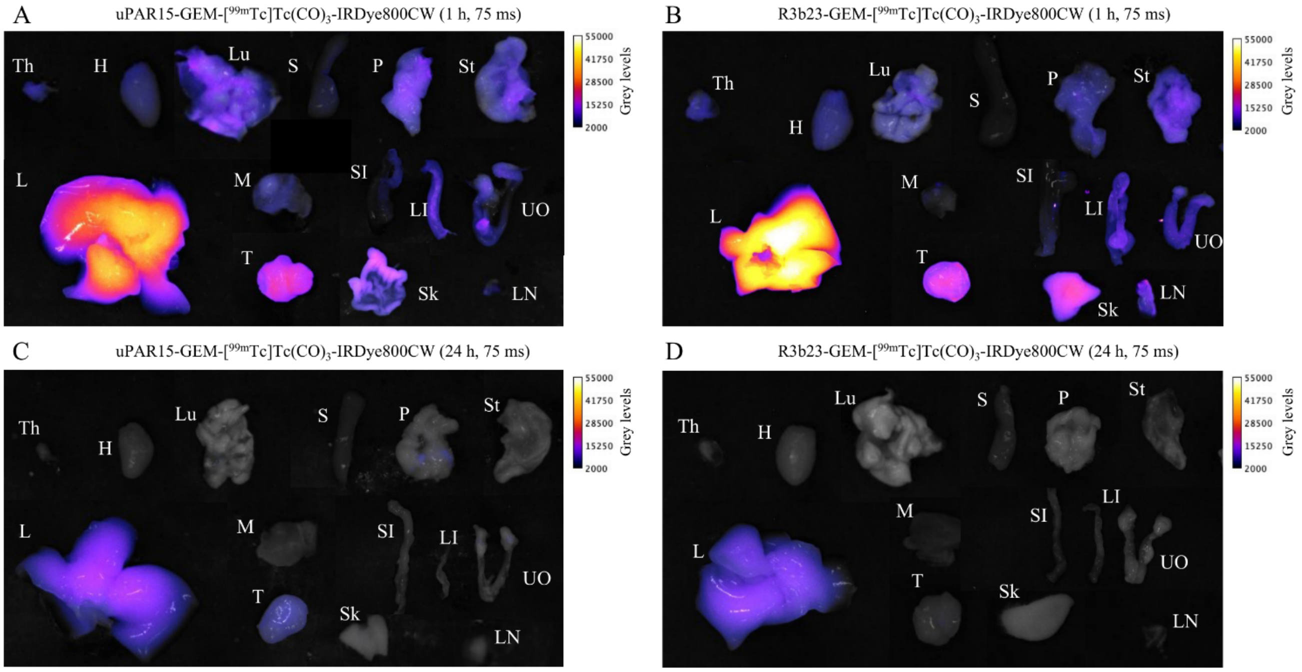

Supplementary Figure 4: 2D fluorescent images of relevant organs and tissues at 1 h (**A**) and 24 h (**C**) post-injection with uPAR15-GEM-[^{99m}Tc]Tc(CO)$_3$-IRDye800CW, and at 1 h (**B**) and 24 h (**D**) post-injection with R3b23-GEM-[^{99m}Tc]Tc(CO)$_3$-IRDye800CW. Thymus (Th), heart (H), lungs (Lu), spleen (S), pancreas (P), Stomach (St), liver (L), muscle (M), small intestines (SI), large intestines (LI), uterus and ovaries (UO), tumor (T), skin (Sk) and lymph nodes (LN) are indicated on the fluorescent images.
